# MiR-223/NFAT5 signaling suppresses arterial smooth muscle cell proliferation and motility *in vitro*

**DOI:** 10.18632/aging.202395

**Published:** 2020-12-28

**Authors:** Feifei Su, Miaoqian Shi, Jian Zhang, Qiangsun Zheng, Haichang Wang, Xue Li, Jianghong Chen

**Affiliations:** 1 Department of Cardiology, Tangdu Hospital, Fourth Military Medical University, Xi’an 710038, Shaanxi, China; 2 Department of Cardiology, Air Force General Hospital, PLA, Beijing 100142, China; 3 Department of Cardiology, PLA Army General Hospital, Beijing 100700, China; 4 Department of Cardiology, Beijing Chest Hospital Heart Center, Capital Medical University, Beijing 101149, China; 5 Division of Cardiology, Second Affiliated Hospital of Xi’an Jiao Tong University, Xi’an 710004, Shaanxi, China

**Keywords:** MiRNA-223, human aortic smooth muscle cells, proliferation, migration, nuclear factor of activated T cells 5

## Abstract

Aberrant proliferation and migration of vascular smooth muscle cells contributes to cardiovascular diseases (CVDs), including atherosclerosis. MicroRNA-223 (miR-223) protects against atherosclerotic CVDs. We investigated the contribution of miR-223 to platelet-derived growth factor-BB (PDGF-BB)-induced proliferation and migration of human aortic smooth muscle cells (HASMCs). We found that miR-223 was downregulated in PDGF-BB-treated HASMCs in a dose- and time-dependent manner, while nuclear factor of activated T cells 5 (NFAT5) was upregulated. Gain- and loss-of-function studies demonstrated that miR-223 treatment reduced PDGF-BB-induced HASMC proliferation and motility, whereas miR-223 inhibitor enhanced these processes. Moreover, NFAT5 was identified as a direct target of miR-223 in HASMC. The inhibitory effects of miR-223 on HASMC proliferation and migration were partly rescued by NFAT5 restoration. Overall, these findings suggest that miR-223 inhibits the PDGF-BB-induced proliferation and motility of HASMCs by targeting NFAT5 and that miR-223 and NFAT5 may be potential therapeutic targets for atherosclerosis.

## INTRODUCTION

Cardiovascular diseases (CVDs) are a group of disorders affecting blood vessels and the heart and are the leading cause of death globally [1]. Atherosclerosis, a chronic inflammatory disease affecting large- and medium-sized arteries, predisposes one to CVDs [2]. Vascular smooth muscle cells (VSMCs) are the major components of the medial layer of aortic blood vessels. The abnormal proliferation and migration of VSMCs with extracellular matrix degradation are critical for the emergence and development of atherosclerosis [3]. At the early stage, VSMCs migrate from the tunica media into the intima of the arterial wall and then massively proliferate, causing intimal thickening, arterial space narrowing, vessel occlusion and ultimately atherosclerosis [3]. Thus, suppressing VSMC motility and proliferation may be an effective therapeutic strategy for atherosclerosis.

Aberrant proliferation and migration of VSMCs can be triggered by various stimuli, especially platelet-derived growth factors (PDGFs) [4, 5]. Among the isotypes of PDGFs, PDGF-BB is the most potent inducer of VSMC pathogenesis due to its ability to bind with all isoforms of the PDGF receptor (PDGFR) [5]. The binding of PDGF-BB to PDGFR activates various signaling pathways which promote the pathogenic transmission of VSMCs [6]. Nuclear factor of activated T cells 5 (NFAT5), a member of the Rel family, is required for PDGF-BB-induced VSMC migration [7]. Although NFAT5 was originally described as a hypertonicity-responsive transcription factor that orchestrates cellular homeostasis [8], it has recently been shown to support the proliferation and migration of multiple cell types [9–11]. However, the biological roles of NFAT5 in PDGF-BB-induced motility and proliferation of VSMCs remain largely unclear.

MicroRNAs (miRNAs) inhibit gene expression by binding to the 3′-untranslated region (3′-UTR) of their target mRNAs [12] and thereby contribute to multiple disorders including CVDs [13, 14]. Several dysregulated miRNAs have been proved to affect the proliferation, migration and apoptosis of VSMCs [15, 16]. Downregulation of miR-22-3p facilitates the pro-proliferative and pro-migratory phenotypes of aortic smooth muscle cells (ASMCs) in arteriosclerosis obliterans [17]. MiR-503 has been reported to inhibit PDGF-BB-induced proliferation and migration of human ASMCs (HASMCs) [18]. MiR-223 restrains hypoxia-caused proliferation, migration, and stress fiber formation in pulmonary ASMCs (PASMCs) [19]. MiR-223 reverses experimental pulmonary arterial hypertension (PAH) *in vivo* and reduces the proliferation and apoptosis resistance of PAH-PASMCs *in vitro*, suggesting that it might affect VSMC function and atherogenesis [19–21]. However, the biological roles of miR-223 in HASMCs are still not fully understood. In this study, we investigated the expression and biological functions of miR-223 in PDGF-BB-exposed HASMCs. Our findings suggest that miR-223 acts as a negative regulator of HASMC proliferation and motility by directly targeting NFAT5, thus providing potential therapeutic targets for atherosclerosis.

## RESULTS

### MiR-223 and NFAT5 are differentially expressed in PDGF-BB-stimulated HASMCs

**Figure 1:**
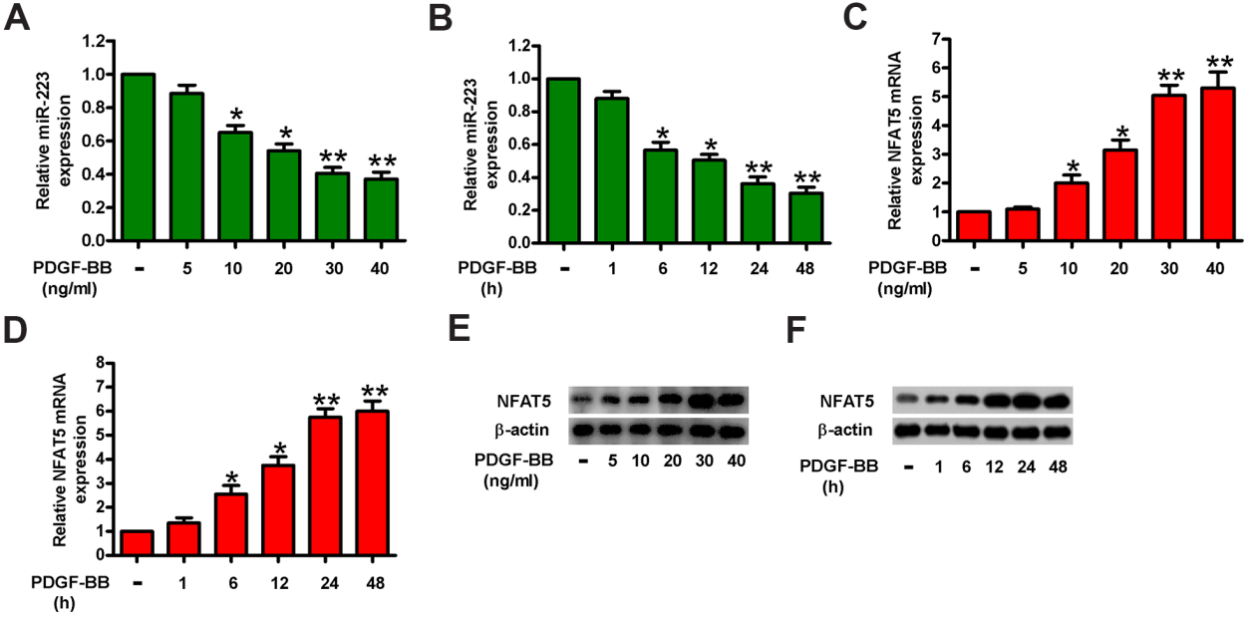
**MiR-223 was increased and simultaneously NFAT5 was decreased in PDGF-BB-treated HASMCs.** HASMCs were starved with 0.5% FBS for 48 h and subsequently exposed to PDGF-BB at various concentrations (5, 10, 20, 30, and 40 ng/ml) for the different time durations (1, 6, 12, 24, and 48 h). (**A**–**D**) miR-223 and NFAT5 mRNA levels were measured by qPCR assays in HASMCs treated with different concentrations of PDGF-BB (**A**, **C**) for the indicated time durations (**B**, **D**). MiR-223 and NFAT5 mRNA expressions were normalized to U6 and GAPDH, respectively. (**E**, **F**) Western blot analyses of NFAT5 expression in HASMCs treated with various doses of PDGF-BB (**E**) for the different time durations (**F**). β-actin was used as the endogenous control. The data are shown as mean ± SD of three separate experiments. ^*^P < 0.05, ^**^P < 0.01 compared with control group.

PDGF is one of the most potent inductors of HASMC proliferation [22]. To examine whether miR-223 and NFAT5 are differentially expressed in proliferated HASMCs, we treated HASMCs with various concentrations (5, 10, 20, 30, and 40 ng/ml) of PDGF-BB for 24 h or with 30 ng/ml of PDGF-BB for the different time durations (1, 6, 12, 24, and 48 h). qPCR assay results revealed that PDGF-BB treatment downregulated miR-223 in HASMCs in a dose- and time-dependent manner (Figure 1A, 1B). Conversely, NFAT5 mRNA (Figure 1C, 1D) and protein (Figure 1E, 1F) levels were augmented in HASMCs upon PDGF-BB stimulation. Treatment with 30 ng/ml of PDGF-BB for 24 h was selected for subsequent experiments due to the considerable decrease in miR-223 and significant increase in NFAT5. These results suggest an inverse correlation between miR-223 and NFAT5 expressions in PDGF-BB-stimulated HASMCs.

### MiR-223 inhibits the proliferation of PDGF-BB-stimulated HASMCs

**Figure 2:**
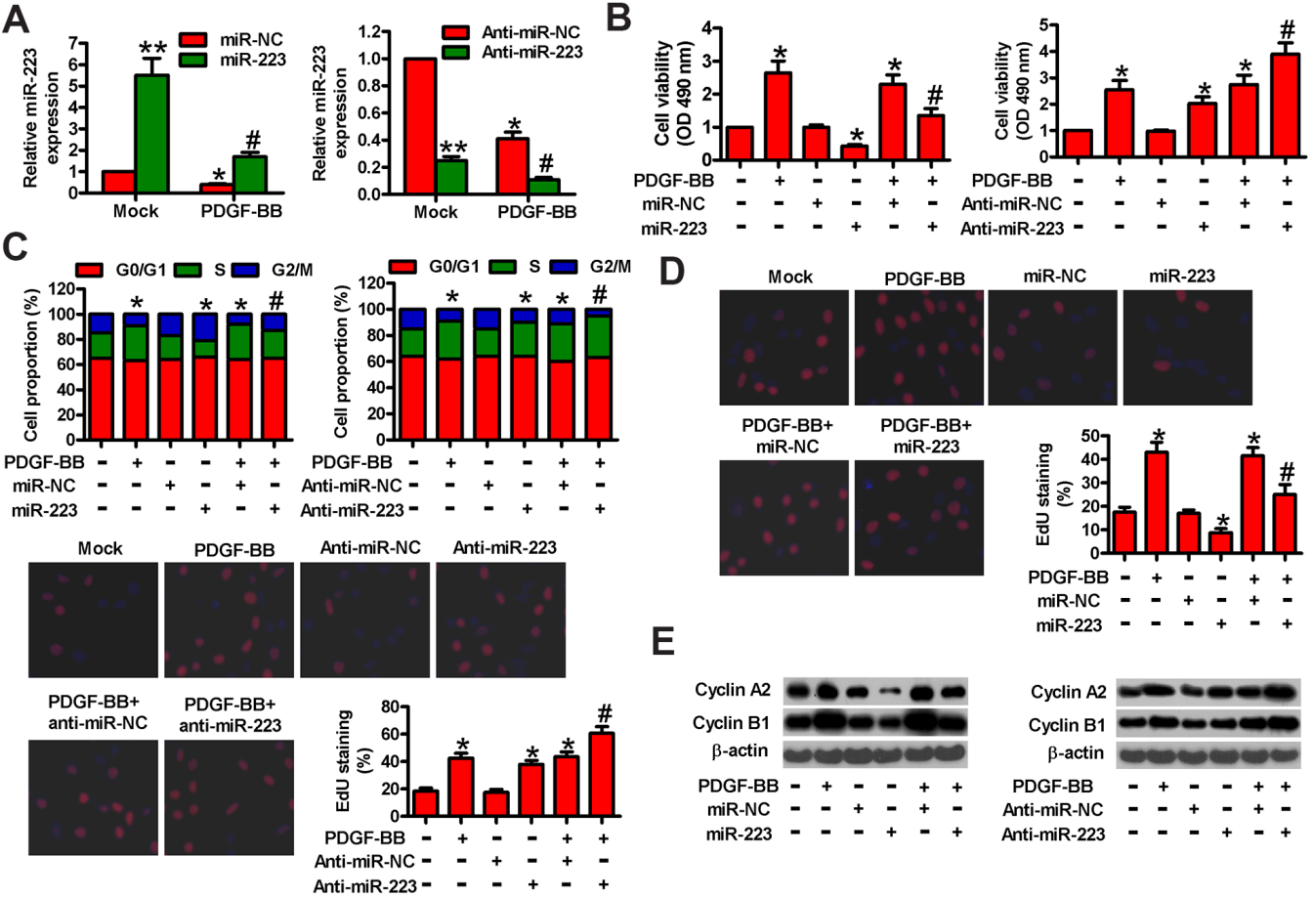
**MiR-223 suppressed PDGF-BB-induced proliferation of HASMCs.** Serum-deprived HASMCs were transfected with miR-223, miR-NC, and anti-miR-223 or anti-miR-NC for 24 h, followed by PDGF-BB stimulation for 24 h. (**A**) miR-223 levels were determined by qPCR assay. MiR-223 expression was normalized to U6. (**B**) MTT assay was performed to measure cell viability. (**C**) Cell cycle distribution was analyzed by flow cytometry. The percentage of cells in G0/G1, S, and G2/M phases were calculated. (**D**) Cell proliferation was assessed by EdU incorporation assay. The percentages of EdU-positive cells were counted. (**E**) Representative Western blot results of cyclin A2 and cyclin B1. β-actin was used as the endogenous control. The data are shown as mean ± SD of three separate experiments. ^*^P < 0.05, ^**^P < 0.01 compared with the miR-NC or anti-miR-NC group in (**A**) and compared with control group in (**B**–**E**). *^#^P* < 0.05 compared with PDGF-BB group.

To investigate the role of miR-223 in PDGF-BB-induced HASMC proliferation, miR-223, miR-NC, anti-miR-223, or anti-miR-NC was transfected into HASMCs. As expected, miR-223 was upregulated in miR-223-transfected HASMCs or downregulated in anti-miR-223-treated HASMCs in the presence or absence of PDGF-BB (Figure 2A). We then performed MTT, EdU incorporation, and flow cytometry assays to determine the effects of miR-223 on cell viability, proliferation, and cell cycle progression. As shown in Figure 2B, miR-223 inhibited the viability of PDGF-BB-treated HASMCs compared with miR-NC-transfected groups. MiR-223 induced G2/M phase arrest in PDGF-BB-exposed HASMCs (Figure 2C). Consistently, miR-223 repressed the PDGF-BB-stimulated proliferation of HASMCs (Figure 2D). However, miR-223 depletion markedly enhanced the viability (Figure 2B) and the S phase proportion (Figure 2C) of PDGF-BB-stimulated HASMCs while increasing their proliferation (Figure 2D). Next, we measured the expressions of two proliferative regulators (cyclin A2 and cyclin B1) that promote S phase and G2/M progression. As shown in Figure 2E, the levels of cyclin A2 and cyclin B1 were increased in PDGF-BB-treated cells and substantially decreased in miR-223-transfected cells. MiR-223 inhibitor caused a further increase of cyclin A2 and cyclin B1 in PDGF-BB-treated cells (Figure 2E). These data indicate that PDGF-BB-induced proliferation of HASMCs is partly suppressed by miR-223.

### MiR-223 represses the motility of PDGF-BB-stimulated HASMCs

**Figure 3:**
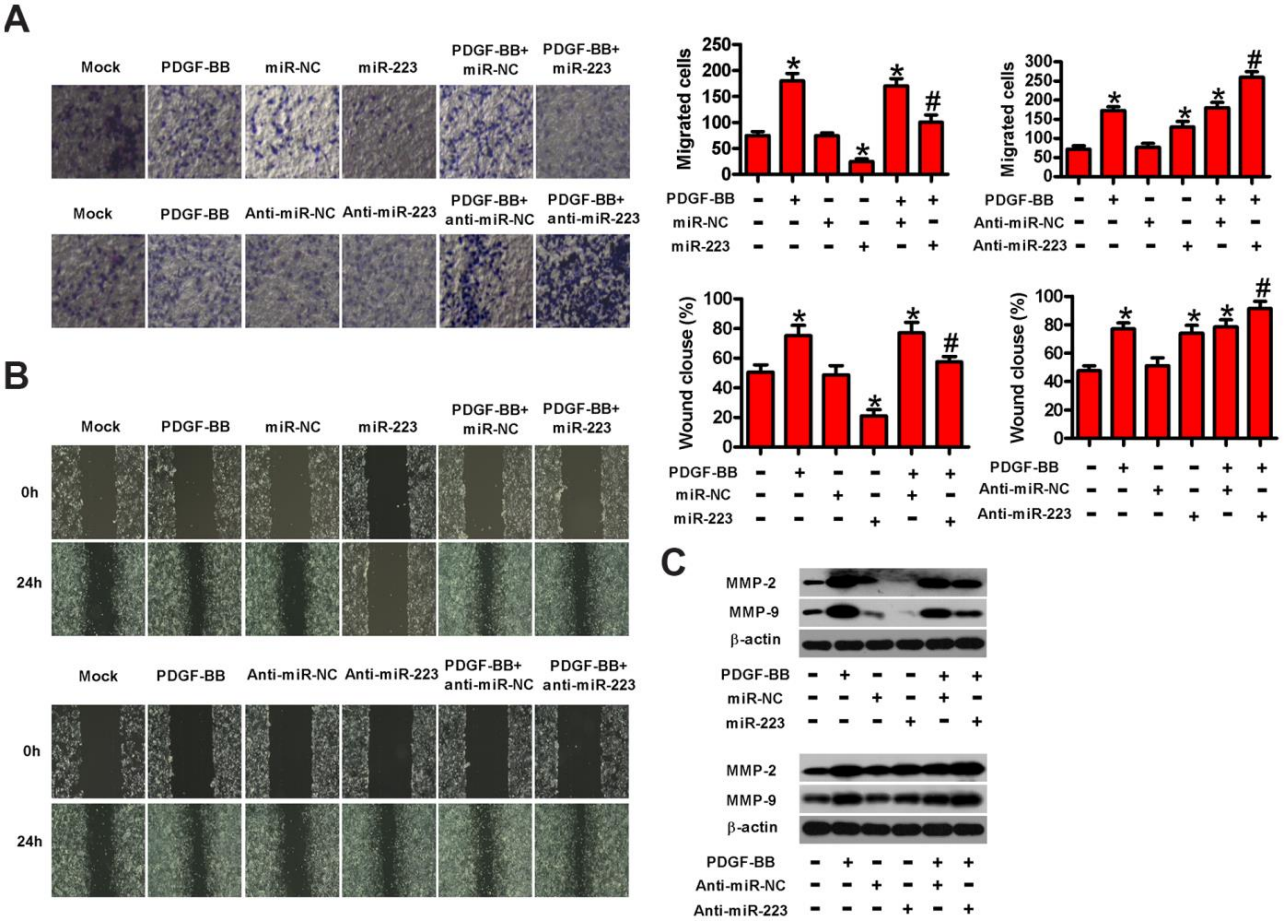
**MiR-223 inhibited the migration of PDGF-stimulated HASMCs.** Serum-deprived HASMCs were transfected with miR-223, miR-NC, and anti-miR-223 or anti-miR-NC for 24 h, followed by PDGF-BB stimulation for 24 h. (**A**) Transwell assay was conducted to assess cell migration. (**B**) The migratory ability of HASMCs was evaluated by wound healing assay. (**C**) Western blot assay was conducted to analyze the expressions of MMP-2 and MMP-9. β-actin was used as the endogenous control. The data are shown as mean ± SD of three separate experiments. ^*^P < 0.05 compared with control group. *^#^P* < 0.05 compared with PDGF-BB group.

To explore whether miR-223 affects the migration of HASMCs, we performed transwell and wound healing assays. Figure 3A shows that miR-223 transfection reduced the PDGF-induced migration of HASMCs compared with controls. Conversely, the downregulation of miR-223 promoted PDGF-induced HASMC migration. Similarly, wound healing assay revealed a decrease in the PDGF-induced migration of HASMCs with miR-223 transfection but the increased migration in anti-miR-223-transfected cells (Figure 3B). At the molecular level, the expressions of MMP-2 and MMP-9, two metastasis-promoting markers, were downregulated after transfection with miR-223, whereas anti-miR-223 treatment elevated MMP-2 and MMP-9 levels in PDGF-stimulated HASMCs (Figure 3C). These results confirm the inhibitory effects of miR-223 on PDGF-induced HASMC migration.

### NFAT5 is a direct target of miR-223 in HASMCs

**Figure 4:**
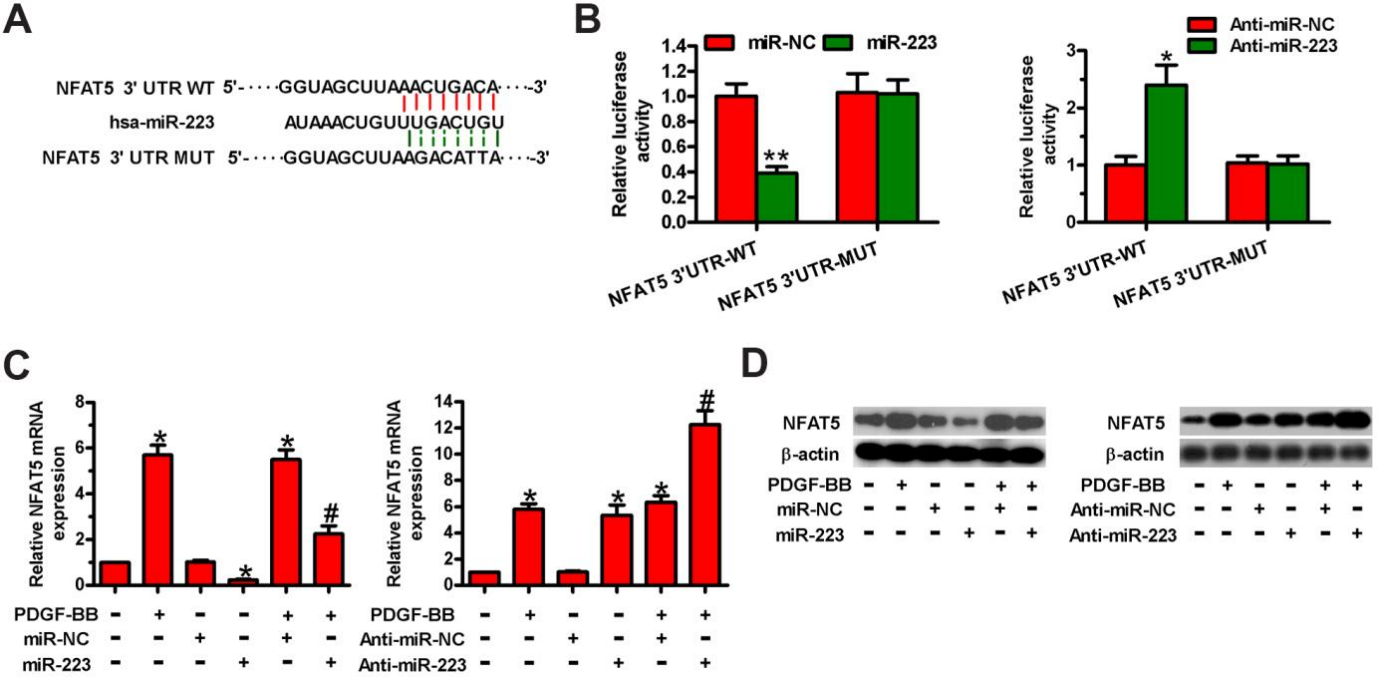
**MiR-223 directly targeted NFAT5 in HASMCs.** (**A**) The putative miR-223 binding sites in the 3′-UTR of NFAT5. (B–D) HASMCs were co-transfected with WT or MUT 3′-UTR of NFAT5 reporter plasmids or miR-223, anti-miR-223, miR-NC, or anti-miR-NC for 48 h. (**B**) Relative luciferase activity was detected. (**C**) qPCR and (**D**) Western blot assays were performed to assess NFAT5 mRNA and protein levels. GAPDH and β-actin were used as the endogenous controls, respectively. The data are shown as mean ± SD of three separate experiments. ^*^P < 0.05, ^**^P < 0.01 compared with the miR-NC or anti-miR-NC group in (**B**) and compared with control group in (**C**, **D**). *^#^P* < 0.05 compared with PDGF-BB group.

Next, we searched for the potential targets of miR-223 by using the TargetScan and miRanda databases. Among the candidates, NFAT5 was identified as a potential target of miR-223 (Figure 4A). We conducted dual-luciferase reporter assay to confirm whether miR-223 directly targets NFAT5. MiR-223 reduced the luciferase activity of the reporter constructs harboring WT 3′-UTR of NFAT5 in HASMCs, whereas the downregulation of miR-223 increased its luciferase activity (Figure 4B). However, neither miR-223 introduction nor depletion had any inhibitory effect on the luciferase activity of the reporter constructs fused to a MUT 3′-UTR of NFAT5 (Figure 4B). Then, we measured the mRNA and protein expressions of NFAT5 in miR-223- or anti-miR-223-transfected HASMCs. The introduction of miR-223 in HASMCs reduced NFAT5 mRNA and protein levels, whereas the knockdown of miR-223 increased them (Figure 4C, 4D). These data demonstrate that NFAT5 is a direct target of miR-223 in HASMCs.

### NFAT5 is involved in miR-223-exerted proliferation inhibition of PDGF-BB- stimulated HASMC

**Figure 5:**
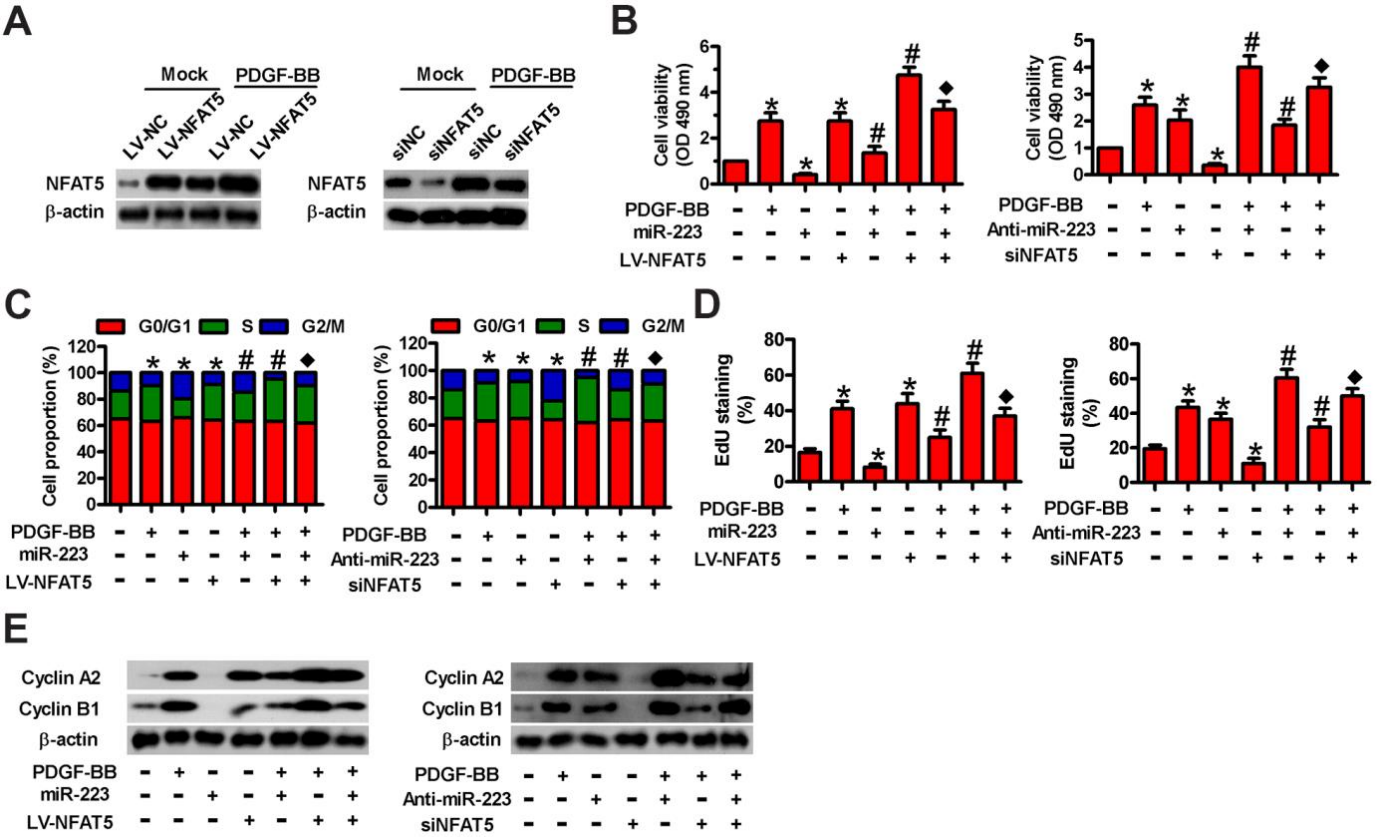
**Involvement of NFAT5 in miR-223-elicited proliferative inhibition of PDGF-BB-exposed HASMCs.** (**A**) Serum-deprived HASMCs were infected with LV-NC or LV-NFAT5 or transfected with siNC or siNFAT5 for 24 h, followed by PDGF-BB stimulation for 24 h. Western blot assay was conducted to detect NFAT5 expression. β-actin were used as the endogenous control. (**B**–**E**) Serum-deprived HASMCs were co-treated with miR-223 and LV-NFAT5 or anti-miR-223 and siNFAT5 for 24 h, followed by PDGF-BB stimulation for 24 h. (**B**) MTT, (**C**) flow cytometry and (**D**) EdU incorporation assays were carried out to analyze cell viability, cell cycle progression and proliferation. (**E**) Representative Western blot results of cyclin A2 and cyclin B1. β-actin was used as the endogenous control. The data are shown as mean ± SD of three separate experiments. ^*^P < 0.05 compared with control group. *^#^P* < 0.05 compared with PDGF-BB group. ^♦^*P* < 0.05 compared with PDGF-BB + miR-223/anti-miR-223 or PDGF-BB + LV-NFAT5/siNFAT5 group.

To probe whether NFAT5 plays a functional role in miR-223-elicited repression of PDGF-BB-induced HASMC proliferation, we carried out MTT, EdU, and flow cytometry assays. We found that LV-NFAT5 infection increased NFAT5 levels in HASMCs with or without PDGF stimulation (Figure 5A), while siNFAT5 transfection reduced them. As depicted in Figure 5B, NFAT5 overexpression counteracted the suppression of cell viability by miR-223 in PDGF-treated HASMCs. Conversely, the increased cell viability by miR-223 inhibitor was reduced by siNFAT5 transfection (Figure 5B). Flow cytometry results showed that miR-223 transfection increased HASMCs at the G2/M phase arrest, whereas anti-miR-223 treatment promoted cell cycle progression of PDGF-stimulated HASMCs (Figure 5C). However, overexpression or knockdown of NFAT5 reversed these alterations (Figure 5C). The reduction or augmentation of proliferation induced by miR-223 introduction or depletion in HASMCs with PDGF stimulation was reversed by NFAT5 overexpression or silencing, respectively (Figure 5D). Molecularly, the miR-223- induced downregulation or anti-miR-223-induced upregulation of cyclin A2 and cyclin B1 in PDGF-stimulated HASMCs was counteracted by NFAT5 overexpression or knockdown, respectively (Figure 5E). These evidences show that NFAT5 is implicated in the miR-223-caused proliferative inhibition of PDGF-BB-treated HASMCs.

### MiR-223 hinders the PDGF-BB-stimulated migration of HASMCs by targeting NFAT5

**Figure 6:**
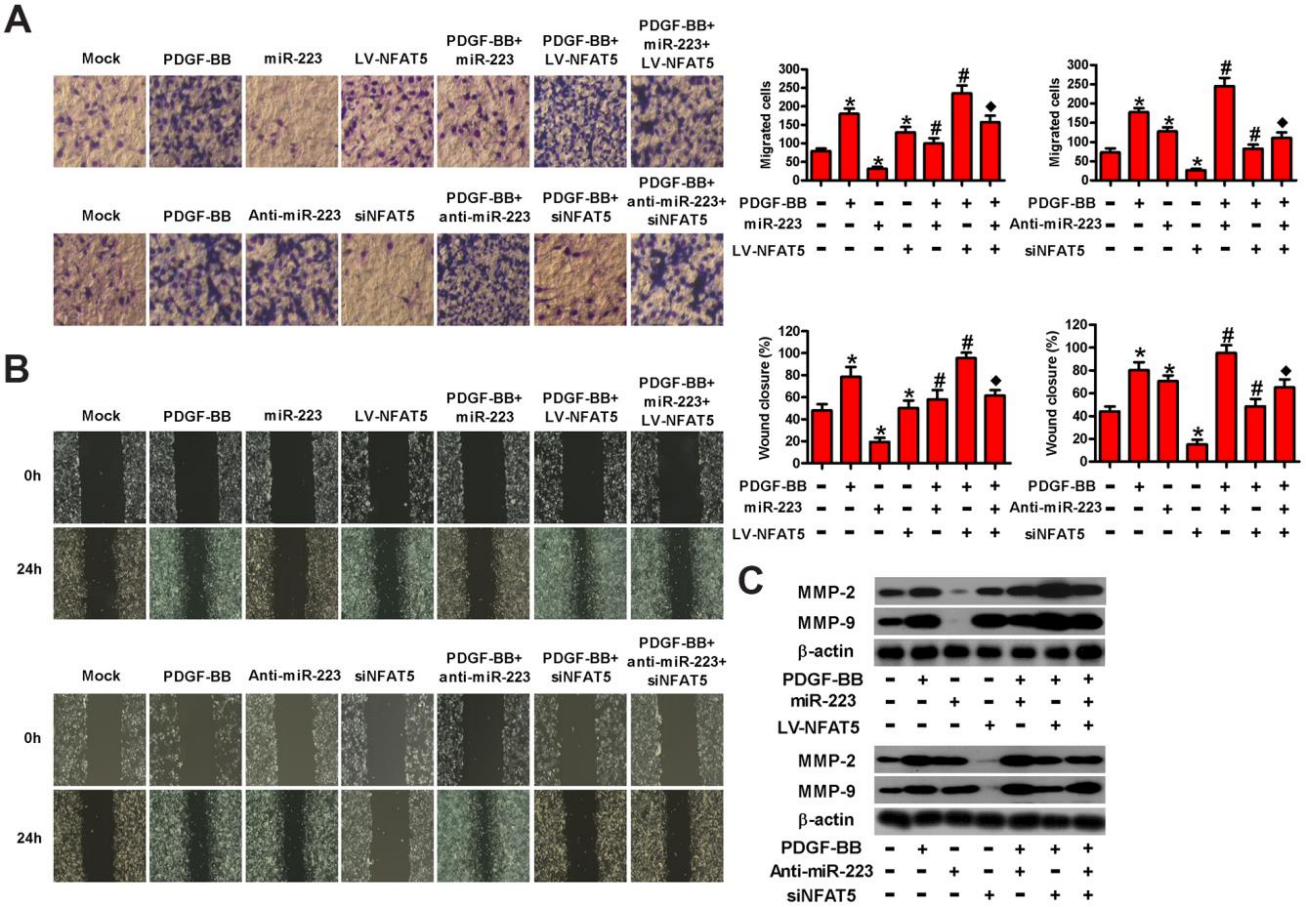
**MiR-223 repressed the migration of PDGF-BB-exposed HASMCs by targeting NFAT5.** Serum-deprived HASMCs were co-treated with miR-223 and LV-NFAT5 or anti-miR-223 and siNFAT5 for 24 h, followed by PDGF-BB stimulation for 24 h. (**A**) Transwell and (**B**) wound healing assays were conducted to measure cell migration. (**C**) The expression of MMP-2 and MMP-9 was detected by Western blot assays. β-actin was used as the endogenous control. The data are shown as mean ± SD of three separate experiments. ^*^P < 0.05 compared with control group. *^#^P* < 0.05 compared with PDGF-BB group. ^♦^*P* < 0.05 compared with PDGF-BB + miR-223/anti-miR-223 or PDGF-BB + LV-NFAT5/siNFAT5 group.

To investigate whether NFAT5 contributed to miR-223-led migration inhibition of PDGF-BB-treated HASMCs, we conducted transwell and wound healing assays. As shown in Figure 6A, LV-NFAT5 infection abated the miR-223-induced repression in the migration of PDGF-BB-exposed HASMCs. However, the miR-223 knockdown- induced increase in the migration of PDGF-BB-stimulated HASMCs was reduced by siNFAT5 transfection. Wound healing assay also demonstrated that NFAT5 overexpression rescued the inhibitory effect of miR-223 on PDGF-BB-induced migration of HASMCs. By contrast, NFAT5 knockdown counteracted the enhanced migratory ability of PDGF-BB-stimulated HASMCs by miR-223 depletion (Figure 6B). The expressions of MMP-2 and MMP-9 were downregulated by miR-223 introduction and were upregulated by miR-223 knockdown in PDGF-BB-stimulated HASMCs, which were reversed by NFAT5 overexpression and silencing, respectively (Figure 6C). These results suggest that NFAT5 conduces to the miR-223-decreased migration of PDGF-BB-treated HASMCs.

## DISCUSSION

In this study, we explored the roles of miR-223 and NFAT5 in the proliferation and motility of PDGF-BB-stimulated HASMCs. We found that miR-223 was downregulated and NFAT5 was upregulated in PDGF-BB-treated HASMCs, and that miR-223 suppressed the proliferation and migration of PDGF-BB-stimulated HASMCs. NFAT5 was identified as a direct target of miR-223 in HASMCs. The inhibitory effects of miR-223 on PDGF-BB-induced HASMC proliferation and motility were elicited by targeting NFAT5. Together, these findings suggest that miR-223 targets NFAT5 to act as a negative regulator of HASMC proliferation and migration.

VSMC proliferation and migration are two hallmarks of atherosclerosis [23]. Some evidences suggest that miRNAs play critical roles in the VSMC proliferation and motility characteristic of atherosclerosis [24, 25]. MiR-22-3p levels are downregulated in arteriosclerosis obliterans tissues and carotid arteries of balloon-injured rats, and miR-22-3p inhibits the proliferation and migration of HASMCs [17]. Liu et al. [26] demonstrated that miR-let-7g suppresses HASMC proliferation and migration *in vitro* and alleviates atherosclerotic mice *in vivo*. MiR-223, a hematopoietic lineage and cell-specific miRNA, has been shown to abrogate PAH progression and repress the proliferation and migration of PASMCs [19–21]. The roles of miR-223 in CVDs have recently drawn increasing attention [27, 28]. MiR-223 released from blood cells protects against vascular neointimal formation and atherosclerosis [29]. In the present study, we found that miR-223 is downregulated in PDGF-BB-stimulated HASMCs. MiR-223 introduction reduced the viability, proliferation, migration, and invasion of PDGF-BB-exposed HASMCs but induced cell cycle arrest at the G2/M phase, coinciding with the downregulation of cyclin A2, cyclin B1, MMP-2, and MMP-9. MiR-223 depletion promoted the proliferation and migration of HASMCs. These gain- and loss-of-function studies show that miR-223 hampers PDGF-BB-induced proliferation and migration of HASMCs, implying that miR-223 affects CVDs.

NFAT5 was originally identified as a potential target of miR-223 in macrophages [30]. Here, we found that NFAT5 is a direct target of miR-223 in HASMCs. NFAT5 is a transcriptional factor that is sensitive to hypertonic stress and regulates gene expression to restore cellular homeostasis [31]. NFAT5 promotes the proliferation of lymphocytes [9] and fibroblast-like synoviocytes [32] and facilitates the migration of skeletal muscle myoblasts [10] and cancer cells [11]. PDGF-BB stimulation increases NFAT5 protein expression and activity in VSMCs, thereby inducing the proliferative or migratory phenotype shifts [7]. NFAT5 contributes to biomechanical stretch-induced inflammation, proliferation, and migration of HASMCs [33]. In this study, we illuminated that NFAT5 mRNA and protein levels are elevated by PDGF-BB stimulation in HASMCs. NFAT5 overexpression markedly attenuated the inhibitory effects of miR-223 on the proliferation and migration of PDGF-BB-treated HASMCs, whereas NFAT5 deficiency had the opposite effects. These findings suggest that miR-223 targets NFAT5 in HASMCs, thereby reducing cell proliferation and migration.

The current study has some limitations. First, we did not investigate the downstream signaling pathways implicated in the anti-proliferative and anti-migratory effects of miR-223 in PDGF-BB-treated HASMCs. Second, we did not explore the mechanism by which PDGF-BB induced miR-223 downregulation. In the future, we will perform *in vivo* studies to further confirm *in vitro* findings.

In summary, miR-223 was downregulated and NFAT5 was upregulated in PDGF-BB-stimulated HASMCs. MiR-223 suppresses the PDGF-induced proliferation and migration of HASMCs by targeting NFAT5. Overall, these results imply that targeting miR-223/NFAT5 axis can be probably exploited for therapeutic benefits to arteriosclerosis.

## MATERIALS AND METHODS

### Cell culture and treatments

HASMCs were purchased from the ScienCell Research Laboratories (San Diego, CA, USA). The cells were cultured in smooth muscle cell growth medium 2 (PromoCell GmbH, Heidelberg, Germany) supplemented with 5% fetal bovine serum (FBS; Gibco, Grand Island, NY, USA) and 50 U/ml of penicillin and 50 μg/ml of streptomycin (both from Sigma-Aldrich, Louis, MO, USA) at 37° C and 5% CO_2_ in a humidified incubator. For PDGF-BB treatment, the cells were starved with 0.5% FBS for 48 h and stimulated with PDGF-BB (R&D, Minneapolis, MN, USA) at different concentrations (5, 10, 20, 30, and 40 ng/ml) for the indicated time durations (1, 6, 12, 24, and 48 h). The introduction or depletion of miR-223 was accomplished by using miR-223 mimics (miR-223), miR-223 inhibitors (anti-miR-223), and their corresponding negative controls (miR-NC and anti-NC; all from RiboBio, Guangzhou, China). For NFAT5 overexpression or knockdown, siRNA targeting NFAT5 (siNFAT5; RiboBio) and the negative control siRNA (siNC) or a lentivirus encoding for NFAT5 (LV-NFAT5; InvivoGen, Hong Kong, China) and the negative control lentiviral vector (LV-NC) were obtained for transfecting or infecting cells. And the sequences used for cell transfection have been listed as follows: for miR-223, 5′-UGUCAGUUUGUCAA AUACCCC-3′; for miR-NC, 5′-GAGCUGGAUGACGAGACCUGU-3′; for anti- miR-223, 5′-AAGACAUUUACAACCUAGAC-3′; for anti-miR-NC, 5′-GGCGAAG GTAGAGTACAGAGA-3′; for siNFAT5, 5′-CAGAGUCAGUCCACAGUUU-3′; for siNC, 5′-GCAUCUGAGUGCAGCUGUC-3′.

### Cell transfection and infection

The oligonucleotides, including miR-223, miR-NC (50 nM), anti-miR-223, anti-miR-NC (100 nM), siNFAT5, and siNC (80 nM), were transfected into the cells using Lipofectamine 2000 (Invitrogen, Carlsbad, CA, USA) according to the manufacturer’s protocol. LV-NFAT5 and LV-NC were infected into the cells at 100 multiplicity of infection.

### Quantitative real-time polymerase chain reaction (qPCR) assay

MiRNAs and mRNAs were isolated from the cells using the miRNeasy Mini Kit and RNeasy kit (both from Qiagen, Hilden, Germany), respectively. The reverse transcription reactions were conducted with a Transcriptor First Strand cDNA Synthesis Kit (Roche, Indianapolis, IN, USA). qPCR assay was performed with SYBR Green PCR Master Mix (Applied Biosystems, Foster City, CA, USA) on a 7500 Real-Time PCR System (Applied Biosystems). U6 and GAPDH were used as endogenous controls. Relative gene expression was calculated using the 2^-ΔΔCt^ method. The primers were as follows: for miR-223, 5′-GGTGTCAGTTTGTCAAATACCC-3′ (forward) and 5′-GTGCAGGGTCCGAGGTCAGAGCCACCTGGGCAATTTTTTT TTTTGG-3′ (reverse); for U6, 5′-GCGCGTCGTGAAGCGTTC-3′ (forward) and 5′- GTGCAGGGTCCGAGGT-3′ (reverse); for NFAT5, 5′-CAGCCAAAAGGGAACTG GAG-3′ (forward) and 5′-GAAAGCCTTGCTGTGTTCTG-3′ (reverse); for GAPDH, 5′-TGTGAACGGATTTGGCCGTA-3′ (forward) and 5′-GATGGTGATGGGTTTCCC GT-3′ (reverse).

### Western blot analysis

The cells were lysed in radio immunoprecipitation assay buffer (Beyotime, Shanghai, China) supplemented with a protease inhibitor cocktail (Roche, Basel, Switzerland). Equal amounts of proteins were separated by sodium dodecyl sulfate polyacrylamide gel electrophoresis and transferred onto polyvinylidene difluoride membranes (Millipore, Bedford, MA, USA). The membranes were blocked with 5% non-fat milk in Tris-buffered saline containing Tween 20 for 1 h and incubated with primary antibodies at 4° C overnight. The primary antibodies were as follows: mouse polyclonal anti-NFAT5 (Abcam, Cambridge, UK), mouse monoclonal anti-cyclin A2, mouse monoclonal anti-cyclin B1, rabbit monoclonal anti-MMP-2, rabbit monoclonal anti-MMP-9, and mouse monoclonal anti-β-actin (all from Cell Signaling Technology, Beverly, MA, USA). The membranes were then incubated with appropriate horseradish peroxidase-conjugated secondary antibodies (Sigma) for 60 min. The protein bands were visualized using an enhanced chemiluminescence kit (Santa Cruz, Dallas, TX, USA) and exposed to X-ray film (Kodak, Fujian, China).

### Cell viability assay

Cell viability was assessed by MTT assay. In brief, HASMCs were seeded in 96-well plates at a density of 1 × 10^4^ cells/well and cultivated for 24 h. After treatment with miR-223, miR-NC, anti-miR-223, anti-miR-NC, miR-223 + LV-NFAT5, or anti-miR-223 + siNFAT5, the cells were incubated with 0.5% FBS for 48 h with or without PDGF-BB (30 ng/ml) stimulation for 24 h after transfection. 200 μl of MTT (5 mg/ml; Sigma) was added to each well and cultured for 4 h; then, 150 μl of dimethyl sulfoxide (Sigma) was added and incubated for 10 min. Absorbance at 490 nm was measured using a microplate reader (Molecular Devices, San Jose, CA, USA).

### Cell proliferation assay

Cell proliferation was measured by EdU incorporation assay. After the cells were subjected to different treatments, EdU (50 μM; RiboBio) was added to the wells and incubated for 24 h at 37° C. The cells were then fixed with 4% paraformaldehyde for 20 min at room temperature and permeabilized with 0.5% Triton X-100 for 10 min, followed by incubation with 1× Apollo reaction cocktail (200 μl) for 30 min. Next, 4′,6-diamidino-2-phenylindole (DAPI; Sigma) was added to the cells for counterstaining. Images were acquired using a fluorescent microscope (Olympus, Tokyo, Japan) and analyzed by Image-Pro Plus 6.0 software (Media Cybernetics, Inc., Rockville, MD, USA). The results are expressed as a percentage of EdU-positive cells.

### Cell cycle analysis

Cell cycle distribution was analyzed by flow cytometry. After the various treatments, the cells were trypsinized, washed with PBS, and fixed with ice-cold 75% ethanol. Subsequently, the cells were incubated with RNase A (20 mg/ml; Sigma) at 37° C for 30 min, stained with propidium iodide (0.5 mg/ml; Sigma) at 4° C for 30 min, and analyzed using a Becton Dickinson flow cytometer (BD Diagnostics, Sparks, MD, USA). The percentages of cells in the G0/G1, S, and G2/M phases were calculated.

### Cell migration assay

The 24-well transwell apparatus with 8 μm pores (Costar, Dallas, TX, USA) was utilized for the migration assay. After transfection or infection, the cells were resuspended in serum-low medium (0.5% FBS) at a density of 1 × 10^6^ cells/ml and seeded into the upper chambers with 200 μl of starved medium. The lower chambers were filled with 500 μl of serum-free medium in the presence or absence of PDGF-BB (30 ng/ml). After cultivation for 24 h, the cells that migrated through the membranes were fixed with 4% formaldehyde (Sigma) and stained with crystal violent (Sigma). Images were taken under a microscope (Olympus), and the number of migrated cells was counted.

### Wound healing assay

After transfection or infection, the cells were seeded in 12-well plates at a concentration of 1 × 10^4^ cells/well and incubated with serum-low medium (0.5% FBS). A straight scratch wound was made using a sterilized 200 μl disposable pipette tip. The cells were then cultured with or without PDGF-BB (30 ng/ml) for 24 h. Images were obtained at 0 and 24 h post-scratch under a microscope (Olympus), and the percentage of wound closure was calculated.

### Dual-luciferase reporter assay

The predicted miR-223 binding site of the wild-type (WT) NFAT5 3′-UTR sequence (5′-TTTGAAGGUAGCUUAAACUGACACCCAT-3′) or mutant (MUT) sequence (5′-TTTGAAGGUAGCUUAAGATATCACCCAT-3′) were amplified and cloned into pmirGLO dual-luciferase vector (Promega, Madison, WI, USA) to create luciferase reporter constructs, namely, pmirGLO-NFAT5-3′-UTR-WT and pmirGLO- NFAT5-3′-UTR-MUT. The constructed plasmids and miR-223, miR-NC, anti-223, or anti-miR-NC or control pRL-TK vector (Promega) were co-transfected into HASMCs using Lipofectamine 2000. At 48 h after transfection, luciferase activity was determined using the Dual-Luciferase Reporter Assay System (Promega) as per the manufacturer’s instructions. Renilla luciferase activity was used as the internal control for normalization.

### Statistical analysis

All the data are expressed as mean ± standard deviation (SD). Statistical analyses were performed using the SPSS 17.0 software (SPSS, Chicago, IL, USA), and the comparisons were made using one-way analysis of variance followed by Tukey’s *post hoc* test. *P* < 0.05 was considered statistically significant.
